# C1-Ten is a PTPase of nephrin, regulating podocyte hypertrophy through mTORC1 activation

**DOI:** 10.1038/s41598-017-12382-8

**Published:** 2017-09-27

**Authors:** Jiyoun Lee, Ara Koh, Heeyoon Jeong, Eui Kim, Tae-Sun Ha, Moin A. Saleem, Sung Ho Ryu

**Affiliations:** 1 Department of Life Sciences, Pohang University of Science and Technology, Pohang, 37673 Republic of Korea; 2 Division of Integrative Biosciences and Biotechnology, Pohang University of Science and Technology, Pohang, 37673 Republic of Korea; 3 Department of Pediatrics, College of Medicine, Chungbuk National University, Cheongju, 28644 Republic of Korea; 4 Academic and Children’s Renal Unit, University of Bristol, Learning and Research, Southmead Hospital, Bristol, BS10 5NB UK

## Abstract

Hypertrophy is a prominent feature of damaged podocytes in diabetic kidney disease (DKD). mTORC1 hyperactivation leads to podocyte hypertrophy, but the detailed mechanism of how mTORC1 activation occurs under pathological conditions is not completely known. Moreover, reduced nephrin tyrosine phosphorylation has been observed in podocytes under pathological conditions, but the molecular mechanism linking nephrin phosphorylation and pathology is unclear so far. In this study, we observed a significant increase in C1-Ten level in diabetic kidney and in high glucose-induced damaged podocytes. C1-Ten acts as a protein tyrosine phosphatase (PTPase) at the nephrin-PI3K binding site and renders PI3K for IRS-1, thereby activating mTORC1. Furthermore, C1-Ten causes podocyte hypertrophy and proteinuria by increasing mTORC1 activity *in vitro* and *in vivo*. These findings demonstrate the relationship between nephrin dephosphorylation and the mTORC1 pathway, mediated by C1-Ten PTPase activity. We suggest that C1-Ten contributes to the pathogenesis of DKD by inducing podocyte hypertrophy under high glucose conditions.

## Introduction

Diabetic kidney disease (DKD) occurs in 20–40% of subjects with both type 1 or type 2 diabetes and is the most frequent cause of end-stage renal disease worldwide^1,2^. DKD is characterized by proteinuria, which contributes to the pathogenesis of progressive renal dysfunction. Proteinuria often results from defects in the glomerular filtration barrier^3–5^. Podocytes are highly specialized and terminally-differentiated cells, and are important as a final barrier to protein during glomerular filtration^6,7^. Thus, podocyte dysfunctions such as foot process effacement, slit diaphragm disruption, podocyte hypertrophy, and cell death have been reported in the early stage of DKD^8–10^. Blockade of podocyte hypertrophy prevents podocyte apoptosis under high glucose conditions^11^; this effect suggests that podocyte hypertrophy precedes podocyte apoptosis, and that inhibition of podocyte hypertrophy induced by high glucose concentration may be a therapeutic target for kidney dysfunction. Recent findings highlight activation of mammalian target of rapamycin complex 1 (mTORC1) as a contributing factor in the development of DKD and podocyte size control^12,13^. However, the key players that regulate mTORC1 activity in diabetes-induced podocyte hypertrophy are poorly understood.

Nephrin is located at the podocyte slit diaphragm^14,15^ and maintains the structural integrity of podocytes by providing a molecular scaffold that links to the actin cytoskeleton^16,17^. Fyn-induced tyrosine phosphorylation of nephrin mediates remodeling of the actin cytoskeleton by providing docking sites for molecules that contain the SH2 domain (e.g., Nck)^18,19^. Nephrin may also function as a signaling regulator because it has a binding site for phosphatidylinositol 3-kinase (PI3K)^20^. However, the function of the PI3K binding site of nephrin in relation to pathology of podocytes is unclear so far. Reduction in nephrin tyrosine phosphorylation has been associated with podocyte dysfunction^21–23^, but the molecular mediators of nephrin tyrosine dephosphorylation such as protein tyrosine phosphatase (PTPase) are much less studied.

C1-Ten (also known as Tensin2) is a member of the tensin family which have an actin binding domain (ABD) and therefore have been suggested to be focal adhesion molecules^24^. Unlike other tensin family, C1-Ten is an active PTPase towards insulin receptor substrate 1 (IRS-1) PI3K binding site^25^. C1-Ten is upregulated in diabetic muscle^25^ and is highly enriched in the glomerulus^26^, especially in the podocytes^27^. These observations suggest that C1-Ten may be a PTPase toward the PI3K binding site of nephrin in the podocytes of kidneys. Because both reduced nephrin tyrosine phosphorylation and increased mTORC1 activation are implicated in podocyte dysfunction, we therefore sought to identify C1-Ten as a PTPase for nephrin in podocytes, and to determine whether this function constitutes a previously-unrecognized step in the progression from nephrin dephosphorylation to mTORC1 activation.

## Results

### C1-Ten PTPase, upregulated by high glucose is responsible for nephrin tyrosine dephosphorylation

**Figure 1:**
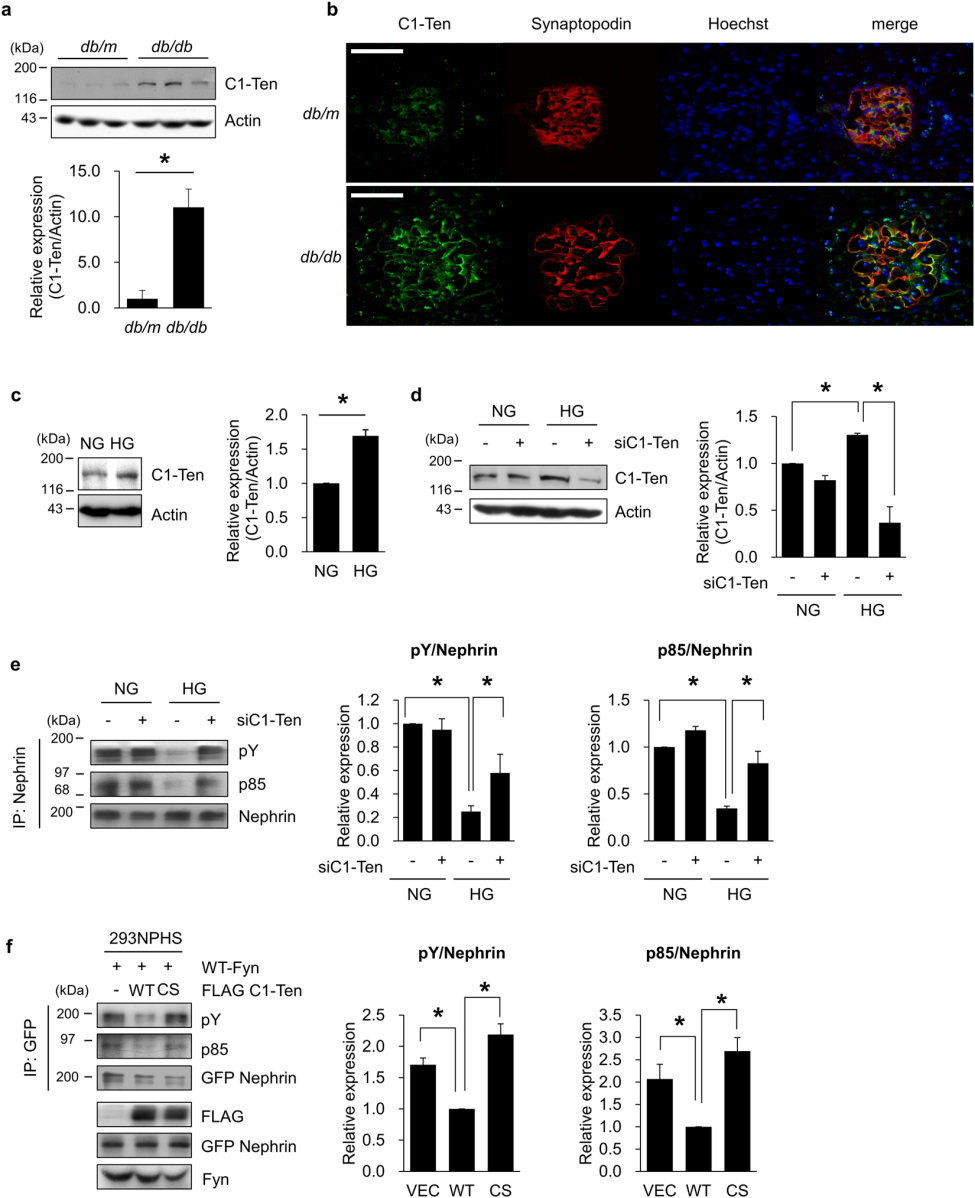
C1-Ten induces dephosphorylation of nephrin in a PTPase-dependent manner. Expression level of C1-Ten in (**a,b**) the kidney of *db/db* mice or (**c**) human podocyte cell line under high glucose (HG) condition. (**a**) Expression levels of C1-Ten were measured by Western blotting in whole kidney lysates of *db/m* and *db/db* mice. Data are means ± SEM (n = 3 mice per group). (**b**) Localization of endogenous C1-Ten was observed in the kidneys of *db/m* and *db/db* mice. Podocyte colocalization was revealed by immunofluorescence of C1-Ten (green), synaptopodin (red) and Hoechst (blue). Original magnification: X400 (scale bar, 50 µm) (**c**) Fully differentiated human podocytes were serum-starved for 18 h, then incubated in medium with normal glucose (NG) or HG for 24 h. Level of C1-Ten protein was measured. Data are means ± SEM (n = 3). (**d,e**) Effect of C1-Ten depletion on the HG-mediated reduction of nephrin phosphorylation. (**d**) C1-Ten knockdown was performed in human podocytes, which were then transfected with 50 nM of control or TNS2 siRNA (siC1-Ten), then stimulated with NG or HG for 24 h. Expression levels of C1-Ten were measured by Western blotting in total cell lysates. (**e**) Remaining cells lysates were subjected to immunoprecipitation with anti-nephrin antibodies. Immunoprecipitates were analyzed by immunoblotting with phosphotyrosine (pY), PI3K regulatory subunit α (p85α), and nephrin antibodies. Data are means ± SEM (n = 3). (**f**) Effect of C1-Ten on Fyn-induced nephrin phosphorylation. 293NPHS cells were cotransfected with WT-Fyn and either FLAG vector, FLAG C1-Ten WT or FLAG C1-Ten CS. GFP nephrin was immunoprecipitated from cell lysates and subjected to immunoblotting with phosphotyrosine (pY), PI3K regulatory subunit α (p85α), and nephrin antibodies. Data are means ± SEM (n = 3). *P < 0.05.

To determine the pathological relevance of C1-Ten, we determined C1-Ten expression in kidneys from mice with type 2 diabetes (*db/db*). C1-Ten expression was significantly elevated in the diabetic kidney (Fig. 1a). C1-Ten was mainly expressed in glomeruli and colocalized with synaptopodin in *db/m* kidneys. The intensity of C1-Ten and its colocalization signal with synaptopodin were greater in *db/db* glomeruli (Fig. 1b). C1-Ten expression was also significantly elevated in kidneys from streptozotocin (STZ)-treated rats compared to control (Supplementary Fig. S1a). High concentration of glucose in the blood is a prominent feature of both type 1 and type 2 diabetes, and of disrupted filtration barrier function of podocytes (Supplementary Fig. S1b). High glucose increased C1-Ten levels in differentiated podocytes (Fig. 1c) and reduced tyrosine phosphorylation and PI3K binding of nephrin (Supplementary Fig. S1c). These results prompted us to assess whether high glucose-induced C1-Ten affects nephrin tyrosine dephosphorylation. C1-Ten depletion in podocytes (Fig. 1d) reversed nephrin tyrosine dephosphorylation induced by high glucose, and the subsequent PI3K binding (Fig. 1e); this result suggests that C1-Ten as a PTPase for nephrin.

Thus, we examined whether C1-Ten regulates nephrin tyrosine dephosphorylation through its PTPase activity. Overexpressed Fyn successfully phosphorylated nephrin in HEK293 cell (Supplementary Fig. S1d). In HEK293 cells expressing functional nephrin (293NPHS cells), C1-Ten WT decreased Fyn-induced nephrin tyrosine phosphorylation and PI3K binding, which was sustained in a C1-Ten phosphatase inactive mutant (CS) (Fig. 1f). These results suggest that enriched C1-Ten in the glomeruli may function as a PTPase towards nephrin.

### C1-Ten activates mTORC1 through its PTPase activity toward nephrin

**Figure 2:**
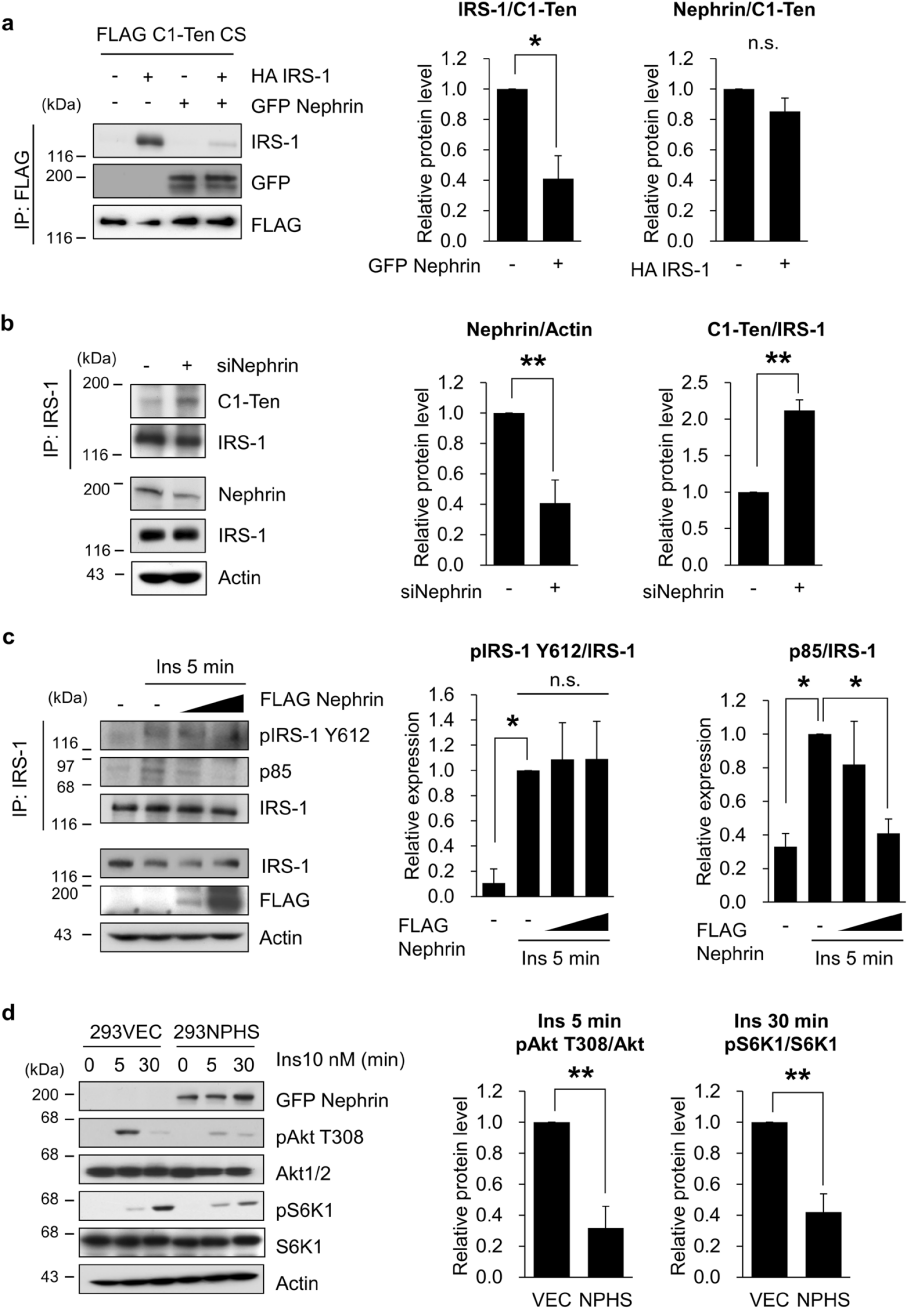
Nephrin competes with IRS-1, sequesters PI3K from IRS-1. (**a,b**) The preferential substrate of C1-Ten when IRS-1 and nephrin coexist. (**a**) FLAG C1-Ten CS was introduced with HA IRS-1 or GFP Nephrin into HEK293 cells, then immunoprecipitated with anti-FLAG beads and immunoblotted with IRS-1, GFP or FLAG. Data are means ± SEM (n = 3). (**b**) Nephrin knockdown was performed in human podocytes, which were transfected with 50 nM of control or NPHS1 siRNA (siNephrin). Cell lysates were subjected to immunoprecipitation with anti-IRS-1 antibodies. Immunoprecipitates were analyzed by immunoblotting with C1-Ten and IRS-1. Expression levels of nephrin, IRS-1 and actin were measured by Western blotting in total cell lysates. Data are means ± SEM (n = 3). (**c**) IRS-1 Y612 phosphorylation and PI3K interaction were monitored by increasing FLAG nephrin. HEK293 cells were transfected with FLAG nephrin. HEK293 cells were serum-starved for 18 h, then treated with 10 nM of insulin for 5 min, and subjected to immunoprecipitation with anti-IRS-1 antibody. The immunoprecipitates were subjected to immunoblotting with pY612 IRS-1, PI3K regulatory subunit α (p85α) or total IRS-1. Data are means ± SEM (n = 3). (**d**) Effect of nephrin on the insulin signaling. 293VEC or 293NPHS cells were serum-starved for 18 h, then treated with 10 nM insulin. Data are means ± SEM (n = 3). *P <0.05; **P <0.01.

Insulin signaling in the podocytes of kidneys contributes to maintenance of structure and function of podocytes and kidneys^28^. Also, C1-Ten acts as a PTPase towards IRS-1 PI3K binding site^25^. Therefore, we tested whether C1-Ten PTPase has a preferential substrate when IRS-1 and nephrin coexist. A mutant with inactive phosphatase has been used to identify its substrate. C1-Ten CS mutant formed a stable complex with nephrin (Fig. 2a); this result suggests that C1-Ten acts as a functional PTPase towards nephrin. C1-Ten CS mutant formed a stable complex with IRS-1, but this interaction was abolished in the presence of nephrin (Fig. 2a). Also, endogenous interaction between IRS-1 and C1-Ten was increased by siRNA-mediated depletion of nephrin (Fig. 2b). These results indicate that in kidney podocytes, C1-Ten acts as a PTPase toward nephrin, but not toward IRS-1.

Because both nephrin and IRS-1 have PI3K binding sites, we determined the effects of nephrin on insulin signaling. Insulin-induced phosphorylation at IRS-1 Y612 (the most important site for PI3K activity) was not affected by nephrin overexpression (Fig. 2c), but the interaction between IRS-1 and PI3K was reduced by nephrin (Fig. 2c); this observation suggests that nephrin sequesters PI3K from IRS-1 and thus might perturb insulin signaling. Indeed, insulin signaling (Akt and S6K1 phosphorylation, downstream of IRS-1) was reduced in the presence of nephrin (Fig. 2d). However, this inhibitory action of nephrin towards insulin signaling can also be contributed by negative feedback towards IRS-1 from nephrin-mediated S6K1 basal phosphorylation (Supplementary Fig. S2)^29^.

**Figure 3:**
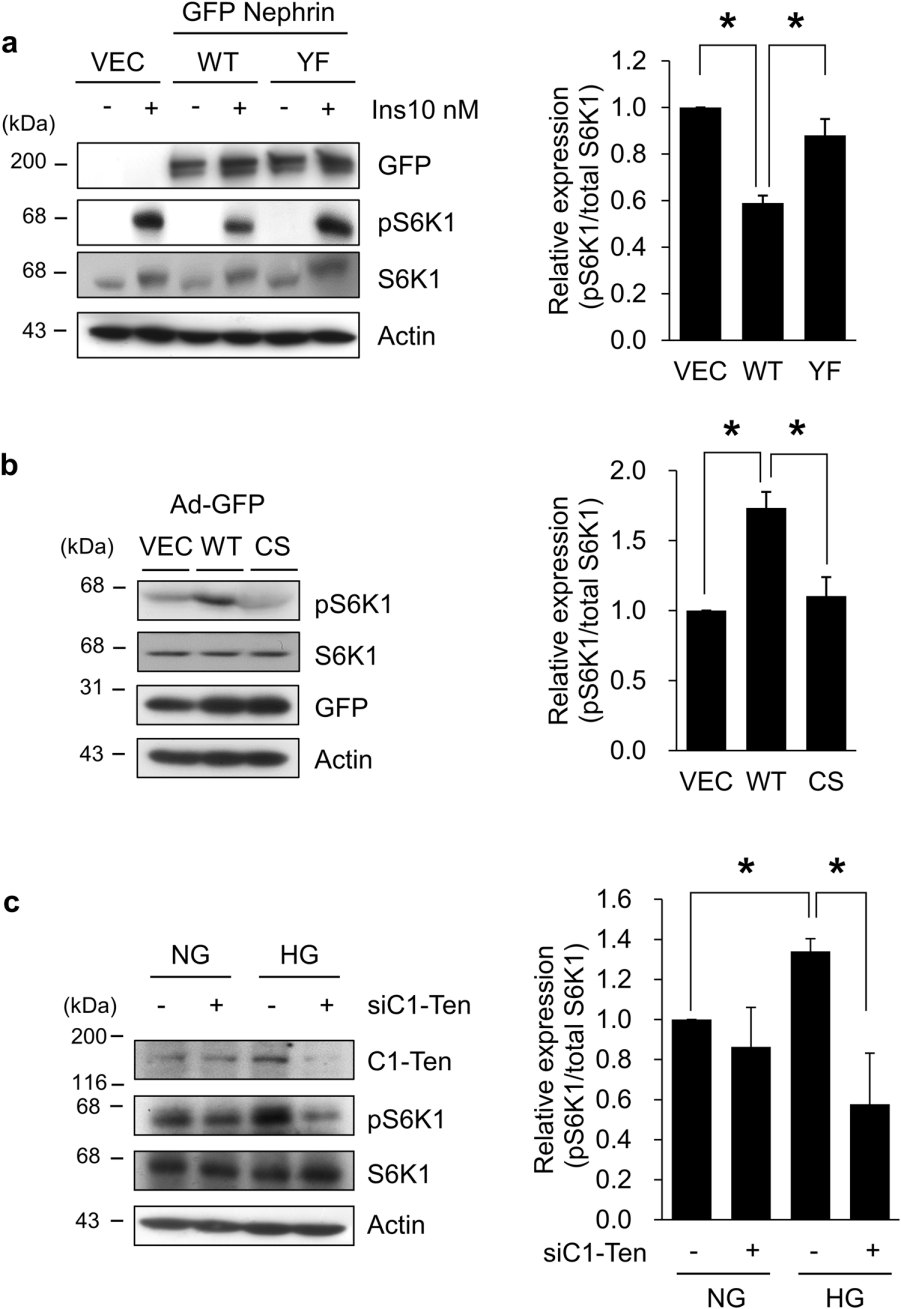
C1-Ten PTPase activates mTORC1. (**a**) Effect of nephrin Y1138F mutant on IRS-1-mediated mTORC1 activation. HEK293 cells were transfected with GFP vector, GFP nephrin WT, or GFP nephrin Y1138F for 24 h. The cells were serum-starved for 18 h, then treated with 10 nM insulin for 30 min. mTORC1 activation was measured by immunoblotting of phospho- and total- S6K1. Data are means ± SEM (n = 3). (**b**) Effect of C1-Ten overexpression on mTORC1 activation. Human podocytes were transduced with Adenovirus (Ad) GFP, Ad C1-Ten WT or Ad C1-Ten CS for 48 h. Protein expression of phospho- and total-S6K1 were detected by Western blotting. Data are means ± SEM (n = 3). (**c**) Effect of C1-Ten knockdown on the HG-mediated mTORC1 activation. mTORC1 activation was presented by immunoblotting of phospho- and total- S6K1. Data are means ± SEM (n = 3). *P <0.05.

Our results indicate that nephrin and IRS-1 compete for PI3K binding (Fig. 2c), so we assumed that reduced nephrin tyrosine phosphorylation will increase the amount of PI3K rendered to IRS-1, and thereby potentiate mTORC1 activation mediated by IRS-1. Indeed, nephrin PI3K binding-deficient mutant (Y1138F) was not efficient in inhibiting insulin-induced S6K1 phosphorylation, compared to those by nephrin WT (Fig. 3a).

Because nephrin dephosphorylation is responsible for mTORC1 activation, and because C1-Ten functions as a PTPase for nephrin, we investigated whether the PTPase activity of C1-Ten activates mTORC1. Adenovirus-mediated C1-Ten expression in differentiated podocytes increased S6K1 phosphorylation (Fig. 3b), but expression of the C1-Ten CS mutant did not increase S6K1 phosphorylation (Fig. 3b); this result is consistent with the hypothesis that C1-Ten PTPase activity regulates mTORC1.

To test whether high glucose-induced S6K1 phosphorylation is mediated by C1-Ten upregulation, we examined the effect of C1-Ten knockdown on S6K1 phosphorylation by using siRNAs against C1-Ten. siRNA-mediated depletion of C1-Ten effectively reduced S6K1 phosphorylation in high glucose conditions (Fig. 3c). Taken together, these results strongly suggest that C1-Ten is a PTPase towards nephrin, and that this function ultimately activates mTORC1 in the podocytes of kidney.

### C1-Ten causes podocyte and kidney dysfunction via its PTPase activity

**Figure 4:**
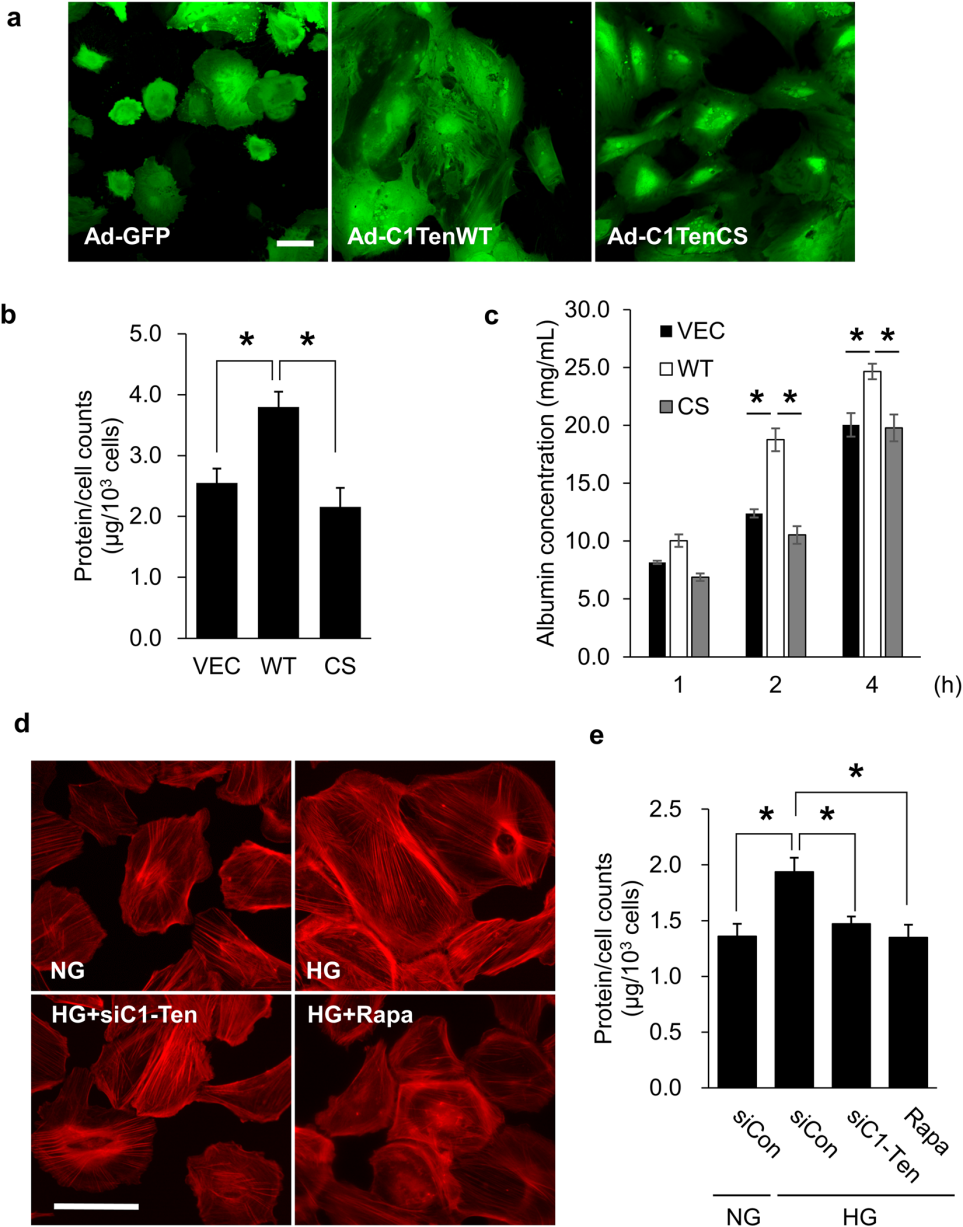
C1-Ten induces podocyte dysfunction in a PTPase-dependent manner. Human podocytes were infected with Ad GFP, Ad C1-Ten WT or Ad C1-Ten CS for 48 h. Podocyte hypertrophy was assessed by (**a**) confocal imaging and (**b**) calculating the amount of protein per cell. Images were obtained by confocal microscopy (Scale bar, 50 µm). Protein/number of podocytes also were evaluated using a hemocytometer and a modified Lowry assay. Data are means ± SEM (n = 3). (**c**) Effect of C1-Ten on albumin influx in transwell permeability assay. Albumin concentration was evaluated by using Bradford assay at various time points. Data are means ± SEM (n = 4). (**d,e**) Effect of C1-Ten depletion on HG-mediated podocyte hypertrophy. Human podocytes were transfected with 50 nM of control or TNS2 siRNA (siC1-Ten), then stimulated with NG or HG for 24 h. Rapamycin (10 µM) was applied as a positive control under HG for 1 h. (**d**) Podocytes were stained with Alexa 594 phalloidin to observe morphology of cells (Scale bar, 100 µm). (**e**) Protein/number of podocytes also were evaluated using hemocytometer and a modified Lowry assay. Data are means ± SEM (n = 3). *P < 0.05.

Because mTORC1 hyperactivation is often related to podocyte dysfunction such as podocyte hypertrophy and proteinuria, we examined the effect of C1-Ten overexpression on podocyte morphology and function. Podocytes infected with Ad C1-Ten WT were significantly enlarged compared with those infected with Ad GFP or Ad C1-Ten CS (Fig. 4a). Podocyte hypertrophy is also evaluated by measurement the ratio of total cellular protein to cell number; the amount of protein per cell was significantly increased in podocytes expressing C1-Ten WT (Fig. 4b). Because podocyte hypertrophy is often associated with loss of filtration function, we used a transwell permeability assay to confirm the effect of C1-Ten on glomerular filtration barrier function of podocytes. Podocytes that were expressing C1-Ten WT showed significantly impaired filtration barrier function with increased albumin leakage (Fig. 4c). In contrast to C1-Ten WT, C1-Ten CS showed no effect on the albumin influx (Fig. 4c), supporting the negative role of C1-Ten in podocyte function via its PTPase activity. We further investigated whether C1-Ten knockdown is sufficient to reverse high glucose-induced hypertrophy of podocytes. C1-Ten knockdown effectively inhibited high glucose-mediated podocyte hypertrophy; the result is the same as the action of rapamycin, which is an mTORC1 inhibitor (Fig. 4d,e). C1-Ten depletion had no additive effect with rapamycin in podocyte hypertrophy mediated by high glucose; this independence suggests that both regulate podocyte size via mTORC1 signaling (Supplementary Fig. 3). These results suggest that the PTPase activity of upregulated C1-Ten exacerbates podocyte hypertrophy and impairs podocyte filtration barrier function by activating mTORC1.

**Figure 5:**
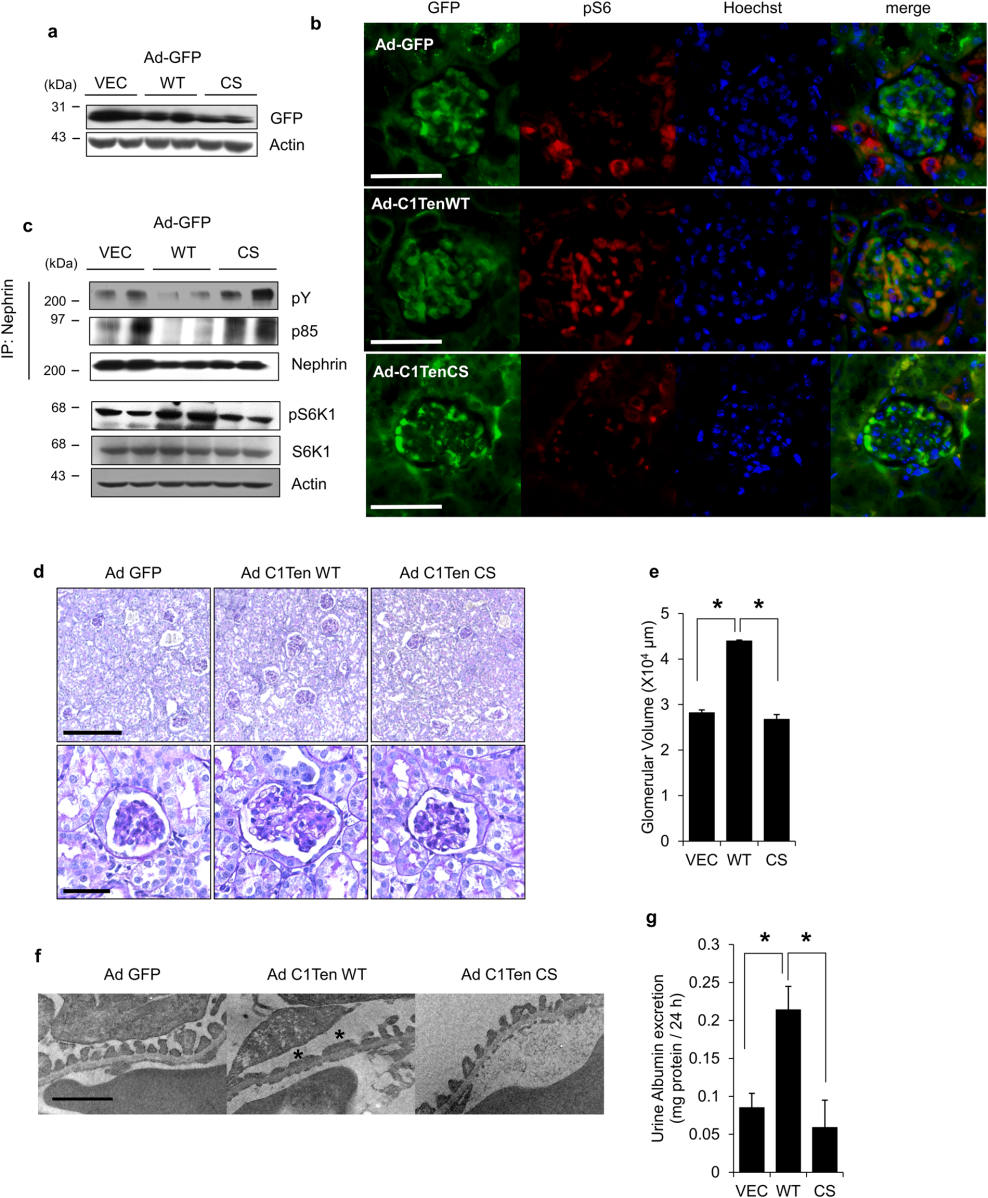
C1-Ten PTPase causes kidney dysfunction *in vivo*. (**a**) GFP expression measured using Western blot analysis of whole kidney lysates. (**b**) Localization of adenovirus-mediated transgene expression. Mice were infected with adenovirus, then sacrificed 7 d later and their kidneys stained with GFP, phospho S6 (red) and Hoechst (blue). Original magnification: X400 (scale bar, 50 µm) (**c**) Effect of C1-Ten overexpression on the nephrin dephosphorylation and mTORC1 activation were measured in adenovirus-infected-whole kidney lysates. Immunoprecipitation was performed with anti-nephrin antibody, and the immunoprecipitates were analyzed by immunoblotting with phosphotyrosine (pY), PI3K regulatory subunit α (p85α), and nephrin antibodies. mTORC1 activation was also measured by immunoblotting of phospho- and total- S6K1. (**d** and **e**) Morphological changes of glomeruli in adenovirus-infected kidneys. (**d**) Adenovirus-infected kidneys stained with periodic acid Schiff (PAS) reagent for renal pathology. Upper image: magnification X100 (scale bar, 100 µm); lower image: magnification X400 (scale bar, 20 µm). (**e**) After PAS staining, glomerular volume was analyzed using Meta Morph image analysis (20 glomeruli per animal). (**f**) Adenovirus-infected kidney podocytes were analyzed by transmission electron microscopy (TEM). TEM analysis revealed effaced podocyte foot process in the kidneys treated with C1-Ten WT (asterisks). Original magnification: X30000 (scale bar, 1 µm) (**g**) Urinary albumin excretion for 24 h was measured using ELISA. Data are means ± SEM (n = 3–5 mice per group). *P <0.05.

**Figure 6:**
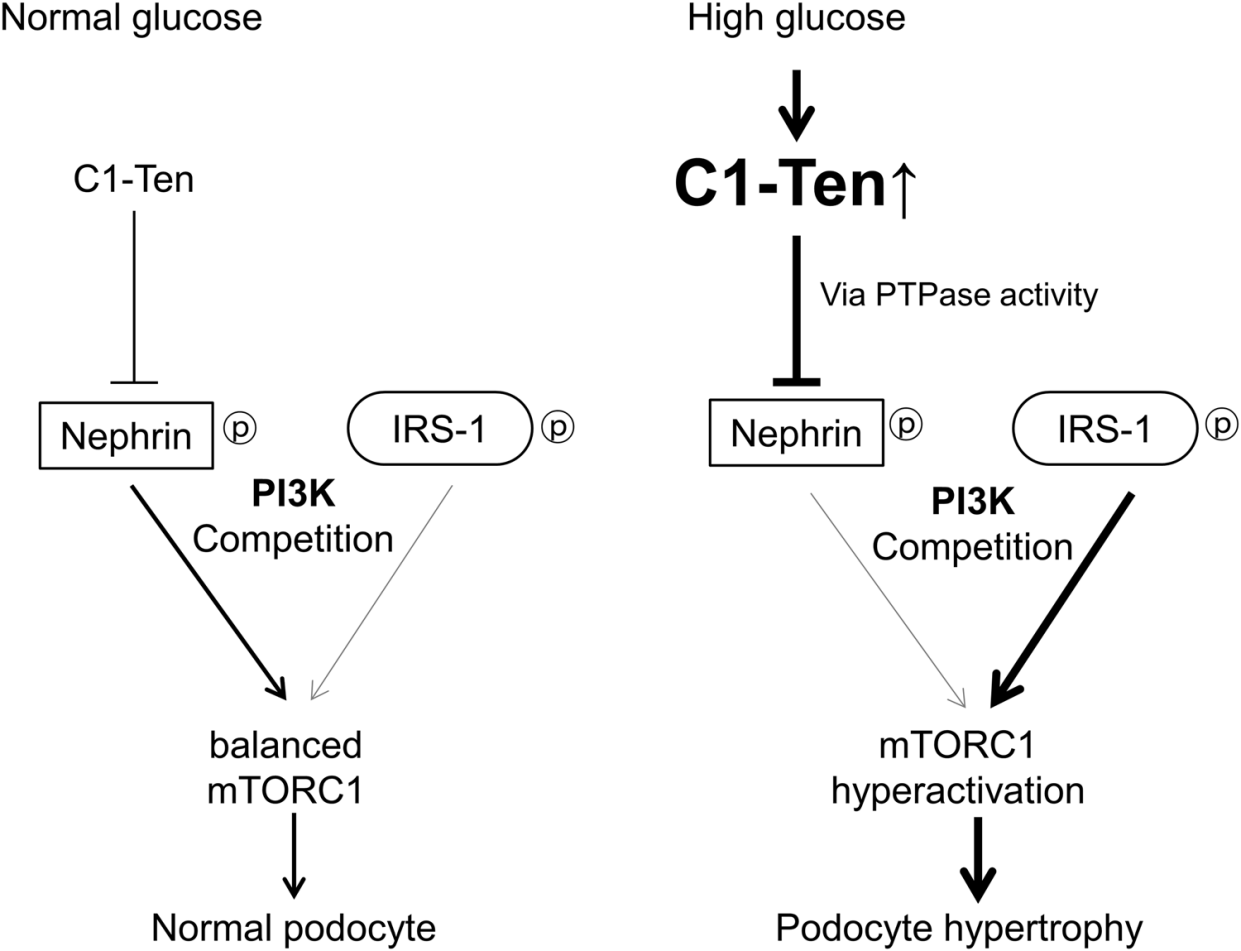
Graphical summary. The balance of mTORC1 activity in normal podocytes is maintained by competition between nephrin and IRS-1 to PI3K. Under high glucose condition, upregulated C1-Ten acts as a PTPase at the nephrin-PI3K binding site and renders PI3K for IRS-1, thereby activating mTORC1. By activating mTORC1, excessive C1-Ten contributes to development of podocyte dysfunctions such as hypertrophy and proteinuria.

Because C1-Ten levels were upregulated in diabetic kidney (Fig. 1a,b) which is characterized by hypertrophy of glomerular cells and elevated urinary albumin secretion, we tested whether C1-Ten affects glomerular size and kidney function *in vivo*. Ad GFP, Ad C1-Ten WT, or Ad C1-Ten CS was introduced to the kidneys by intrarenal injection; 7 d later GFP expression was detected in the kidneys (Fig. 5a). We observed that adenoviral-mediated GFP was expressed throughout the kidneys, including in glomeruli (Fig. 5b). C1-Ten expression in kidney was enough to activate mTORC1, which was also dependent on C1-Ten PTPase activity (Fig. 5c). Also, S6 phosphorylation was increased in glomeruli treated with Ad C1-Ten WT (Fig. 5b). Moreover, the observation that regulation nephrin phosphorylation by C1-Ten catalytic sites (Fig. 5c) suggests that C1-Ten acts as a PTPase toward nephrin *in vivo*. The ratio of kidney/body weight was significantly larger in mice treated with Ad C1-Ten WT than in mice treated with Ad GFP or Ad C1-Ten CS (Table 1). Also, glomerular volume was larger in kidneys treated with C1-Ten WT than in kidney treated with Ad GFP or Ad C1-Ten CS (Fig. 5d,e). Electron microscopy revealed that the podocytes with Ad C1-Ten WT showed focal foot process effacement (Fig. 5f). Moreover, the increase in urinary albumin excretion in a PTPase activity-dependent manner demonstrates that C1-Ten impaired podocyte barrier function *in vivo* (Fig. 5g). However, C1-Ten expression in kidney did not affect glucose levels (Table 1). These results suggest that C1-Ten acts after glucose production is elevated, but before the kidney hypertrophies and glomerular permeability increases. Together, these results suggest that C1-Ten PTPase may negatively regulate kidney function through the C1-Ten/nephrin/mTORC1 axis (Fig. 6).

**Table 1:** Characteristics of animals.

	**Adenovirus**		
	Ad GFP	Ad C1-Ten WT	Ad C1-Ten CS
Blood glucose (mg/dL)	152 ± 12	130 ± 3	148 ± 14
Body weight (g)	21.4 ± 0.4	20.4 ± 0.2	19.6 ± 0.5*
Kidney weight (g)	0.188 ± 0.004	0.193 ± 0.004	0.166 ± 0.002*^†^
Kidney-to-body ratio (mg/g)	0.854 ± 0.007	0.941 ± 0.018*	0.848 ± 0.019^†^

Data are mean ± SEM of n = 3–5 mice per group. *P < 0.05 vs Ad GFP; ^†^P < 0.05 vs Ad C1-Ten WT.

## Discussion

Reduced nephrin tyrosine phosphorylation is implicated in the podocyte pathology of DKD, but the link between nephrin dephosphorylation and podocyte dysfunction at the molecular level, and the molecule responsible for mediating this event, have not been reported. Here we show that the PTPase activity of C1-Ten towards nephrin links caused reduction in nephrin tyrosine phosphorylation, which ultimately led to increased mTORC1 activity in the pathology of podocytes and kidney. We have suggested that nephrin tyrosine phosphorylation may prevent mTORC1 hyperactivation by sequestering PI3K from IRS-1.

Reduction of nephrin tyrosine phosphorylation was observed in various pathological conditions such as puromycin aminonucleoside (PAN) nephrosis^21^, angiotensin II stimulation^22^, and diabetes^23^. Nephrin is tyrosine phosphorylated by the Src-family kinase Fyn; massive proteinuria and morphological changes are observed in Fyn-kinase-knockout mice^18^. Reduced nephrin phosphorylation may induce podocyte damage by remodeling the actin cytoskeleton as a consequence of Nck1/2 binding^19^. Progressive proteinuria develops with structural changes in the filtration barrier in nephrin mutant knockin mice with mutations of the three tyrosine phosphorylation sites for Nck1/2 binding^30^. These findings strongly suggest that nephrin dephosphorylation is important in the pathological process of podocytes; however, the regulation mechanism by which PTPase dephosphorylates nephrin is relatively unexplored. PTP1B is upregulated in a rat PAN nephrosis model, and suppresses nephrin tyrosine phosphorylation; this suppression may result in impaired regulation of the actin cytoskeleton in podocytes^31^. In diabetic Ins^2+/C96Y^ mice, high glucose level upregulates SHP1 in cultured podocytes; this process reduces nephrin phosphorylation and leads to podocyte dysfunction and apoptosis^23^. Phosphorylated nephrin may interact with the PI3K regulatory subunit α (p85α)^20,32^, which is involved in a variety of cellular responses including podocyte survival^23,33^. However, no relationship between PI3K binding site and other podocyte dysfunction has been reported. Also, the PTPase that affects the nephrin-regulating PI3K binding site has not been reported. In this study, we evaluated whether C1-Ten is a PTPase of nephrin, and whether C1-Ten contributes to regulation of PI3K binding and ultimately to podocyte hypertrophy.

Podocyte hypertrophy is a main symptom of DKD. mTORC1 hyperactivation drives podocyte hypertrophy and dysfunction. mTORC1 is highly activated in podocytes of both type 1 and type 2 diabetes^12,13^, and is associated with severe podocyte dysfunctions such as podocyte dedifferentiation, foot process effacement, podocyte loss, and proteinuria in mice from which podocyte-specific Tsc1 had been knocked out^12^. Also, podocyte-specific genetic deletion of mTORC1 (podocyte-specific Raptor knock-out) effectively enhanced podocyte functions and morphology under pathological condition but impaired podocyte function under physiological condition^13^. These results indicate that balanced activation of mTORC1 is important in podocyte function. Nephrin contributes to mTORC1 activation in a PI3K-dependent manner under basal conditions^29^, but no study has assessed whether nephrin tyrosine dephosphorylation participates in mTORC1 activation and podocyte hypertrophy under pathological conditions. In this study, we suggested that nephrin dephosphorylation might be important to regulate mTORC1 activity under high glucose conditions. We provided data that mTORC1 signaling is elevated with nephrin Y1138F mutant compared to nephrin WT; this result suggests that nephrin dephosphorylation may directly regulate mTORC1 signaling (Fig. 3a). We suggest that C1-Ten is a PTPase of nephrin, and regulates PI3K binding of nephrin (Fig. 1e,f), thereby mediating podocyte hypertrophy (Fig. 4) in cultured podocytes. Consistent with these observations, C1-Ten induced mTORC1 activity in a PTPase-dependent manner (Fig. 3b). These data demonstrated that C1-Ten-mediated regulation of nephrin phosphorylation is important to control podocyte hypertrophy under pathological conditions. These results clearly suggest that C1-Ten affects the PI3K binding site of nephrin, and that nephrin phosphorylation on Y1138 is important to regulate mTORC1 signaling, but whether C1-Ten specifically affects the PI3K binding site of nephrin remains unknown. Because other tyrosine sites of nephrin are also involved in podocyte dysfunction, the effect of C1-Ten on the other tyrosine sites of nephrin should be identified. Also, detailed identification of the mechanism by which nephrin phosphorylation affects mTORC1 signaling will require further study.

In this study, we demonstrated that overexpressed C1-Ten mediated kidney dysfunction including albuminuria and glomerular hypertrophy in DBA/2 strain via PTPase activity (Fig. 5). Several studies also reported the effect of C1-Ten on podocyte and kidney function. The *Tns2* gene that encodes C1-Ten is mutated in the ICR-derived glomerulonephritis (ICGN) mouse: a deletion mutant causes a frameshift that produces a terminal codon at a premature position^34^. The ICGN mouse, an animal model for nephrotic syndrome, shows severe proteinuria and glomerulosclerosis with impairment of nephrin and synaptopodin^35^. Also, podocyte abnormalities and proteinuria are observed in mice with C1-Ten SH2-PTB domain deletion mutant^36^. These results and our study suggest that both C1-Ten WT overexpression and C1-Ten mutation contribute to podocyte dysfunction and proteinuria. To understand more precise role of C1-Ten, kidney and podocyte function should be demonstrated in kidney-specific or podocyte-specific inducible conditional KO mouse. Additionally, ICGN mice develop nephrotic syndrome, depending on the genetic background; DBA/2J and FVB/N background are susceptible to glomerulosclerosis, whereas C57BL/6J and 129/Sv strains are resistant to glomerulosclerosis^37–39^. Like in ICGN mouse studies, C1-Ten overexpression might show various severity of kidney dysfunction in other genetic backgrounds, so further study is required.

C1-Ten is a member of the tensin family, which has an ABD that is involved in cytoskeleton reorganization^24^. Among the tensin family, only C1-Ten has a phosphatase catalytic motif and a PTEN-like activity in cells^40,41^. Recently, pathological functions of C1-Ten have been suggested, including diabetes. In diabetic muscle, mRNA and protein level of C1-Ten is significantly upregulated and activates the catabolic pathway by reducing IRS-1 stability in a manner that is related to PTPase activity^25^. Here, we also demonstrate that C1-Ten expression is elevated in diabetic kidneys and in podocytes cultured under high glucose conditions (Fig. 1a–c). Upregulated C1-Ten seems to accelerate diabetes-mediated pathological features including muscle atrophy and podocyte hypertrophy by affecting IRS-1 in muscle but nephrin in podocytes. Thus, the substrates of C1-Ten should be identified in different cell types and tissues. Also, our study indicates that targeting C1-Ten PTPase has a therapeutic potential for diabetes-associated symptoms. In summary, our study demonstrates that nephrin dephosphorylation by C1-Ten activates mTORC1 under high glucose conditions, and thereby contributes to the progression of podocyte hypertrophy. We suggest that inhibition of C1-Ten PTPase activity may alleviate symptoms of DKD.

## Materials and Methods

### Antibodies and reagents

All chemicals were obtained from Sigma-Aldrich (ST. Louis, MO, USA) unless otherwise stated. Anti-C1-Ten (HPA034659) for immunohistochemistry was purchased from Sigma-Aldrich. Anti-C1-Ten (#11990), anti-phospho-Akt T308 (#9275), anti-phospho-S6K1 T389 (#9205), and anti-S6K1 (#9202) antibodies were purchased from Cell Signaling Technology (Danvers, MA, USA). Anti-IRS-1 (#06-248) and anti-phospho-IRS-1 Y612 (#09-432) antibodies were acquired from Millipore (Darmstadt, Germany). Anti-Akt1/2 (sc-1619), anti-pY20 (sc-508), anti-pY99 (sc-7020), anti-synaptopodin (sc-21537), anti-GFP (sc-9996) and polyclonal anti-nephrin (sc-28192) antibodies were obtained from Santa Cruz Biotechnology (Santa Cruz, CA, USA).

### Cell culture

Conditionally immortalized human podocytes were used as previously described^42^. In brief, podocytes were maintained and propagated at 33 °C in RPMI-1640 medium supplemented with 10% (vol/vol) fetal bovine serum (FBS, Gibco, Life Technologies, Carlsbad, CA, USA), with insulin, transferrin, and selenite. To induce differentiation, cells were cultured under growth-restricting conditions at 37 °C for 10–14 d. Fully-differentiated podocytes were serum-deprived for 18 h in a medium containing 5 mmol/L D-glucose before being exposed to various experimental conditions. Podocytes were incubated in cultured medium containing either 5 mmol/L (normal glucose, NG) or 30 mmol/L glucose (high glucose, HG). For C1-Ten knockdown experiments, podocytes were transfected with either 50 nM of negative-control small interfering RNA (siRNA) (Con si) or TNS2 siRNA (siC1-Ten) (Bioneer, Daejeon, South Korea) using Lipofectamine RNAiMAX (Invitrogen, Carlsbad, CA, USA) for 24 h. For nephrin depletion experiments, podocytes were transfected with either 50 nM of Con si or NPHS1 siRNA (siNephrin) by using Lipofectamine RNAiMAX (Invitrogen) for 48 h. For overexpression of C1-Ten in podocytes, the fully differentiated podocytes were infected with adenoviruses for 48 h.

HEK293 cells were grown and maintained in high glucose Dulbescco’s modified Eagle’s medium (DMEM, Lonza, Basel, Switzerland) with 10% (vol/vol) FBS. The stable cell line of 293NPHS cells expressing a GFP-tagged human full-length nephrin construct, was selected in medium containing 1 mg/mL Geneticin. cDNA transient transfection was performed using Lipofectamine (Invitrogen) according to the manufacturer’s instructions.

### Plasmid constructs

FLAG C1-Ten WT and FLAG C1-Ten C231S were generated from the full-length coding region of human C1-Ten (TNS2 isoform 2, KIAA1075) cDNA as described previously^25^. A GFP-tagged full-length human nephrin-pcDNA3 was provided by Dr. Alessia Fornoni (University of Miami, FL, USA). WT-Fyn was a gift from Dr. Puneet Garg (University of Michigan, Ann Arbor, MI, USA).

### Assessment of hypertrophy in cultured podocytes

Hypertrophy of cultured podocytes was evaluated by calculating the amount of protein per cell. Fully differentiated podocytes were infected with GFP-expressing adenovirus for 48 h, then harvested by trypsinization, pelleted at 1,500g for 5 min, and resuspended in PBS. Podocyte cell counts were performed by using hemocytometer. Remaining cells were lysed in 0.5 mol/L NaOH and total protein was analyzed using a modified Lowry method.

### Transwell permeability assay

Podocytes were seeded and grown to confluence in the top chamber of collagen-coated 0.4-μm transwell filters (#3493, Corning Life Sciences, Corning, NY, USA). After podocytes had fully differentiated, they were exposed to various experimental conditions and serum-deprived for 18 h. Then the top chamber was filled with serum-free medium and the bottom chamber was filled with 40 mg/mL of bovine serum albumin, then incubated at 37 °C. Aliquots of medium from the upper chamber were collected at various time points. Albumin concentration was measured using a Bradford assay.

### Animals

All animal experiments were approved by the Pohang University of Science and Technology Institutional Animal Care and Use Committee (POSTECH IACUC) (Approval no. POSTECH-2014-0006). All animal experiments were performed in our animal facility under the IACUC guidelines and regulations. Twelve-week-old male *db/db* mice and male non-diabetic control *db/m* mice (Central Lab Animal Inc, Seoul, Korea) were purchased and sacrificed to evaluate the expression level of C1-Ten in type 2 diabetic kidneys. To elucidate the pathological role of C1-Ten in kidneys, adenovirus was administered to 8-week-old male DBA/2 mice (Central Lab Animal Inc). Adenovirus (Ad) C1-Ten WT and Ad C1-Ten CS were generated and purified as described previously^25^. Ad GFP was used as control. *In vivo* intrarenal virus transduction was performed as described earlier^43–45^. Animals that had received Ad GFP, Ad C1-Ten WT or Ad C1-Ten CS were sacrificed after 7 d. Urine was collected during 24 h for protein assays. Urinary albumin was analyzed using a commercial ELISA kit (Abcam) following the manufacturer’s instructions.

### Histological analysis

Kidneys were removed from DBA/2 mice receiving adenovirus, then fixed with formalin and embedded in paraffin. In each mouse, quantitative analysis of glomerular volume stained with periodic acid Schiff (PAS) reagent was performed as described previously^46^ by using MetaMorph imaging software for microscopy (Universal Imaging, West Chester, PA, USA).

### Immunofluorescence

Formalin-fixed sections were stained with antibodies against GFP with secondary Alexa Fluor 488 donkey anti-mouse antibody (Invitrogen-Molecular Probes, Eugene, OR, USA). Kidneys of *db/m* or *db/db* mice were embedded in a FSC 22 frozen section media (Leica Biosystems, Richmond, IL, USA) for dual immunofluorescence. Frozen sections were stained with C1-Ten and synaptopodin followed by secondary Alexa Fluor 488 donkey anti-rabbit antibody and secondary Alexa Fluor 594 donkey anti-Goat antibody (Invitrogen-Molecular Probes). Podocytes were grown and differentiated on collagen-coated coverslips, infected with GFP-expressing adenovirus for 48 h, then fixed with 4% paraformaldehyde for 30 min at 37 °C. Podocytes with siC1-Ten or rapamycin were stained with Alexa Fluor 594 phalloidin (Invitrogen-Molecular Probes) to observe morphology of cells. The coverslips were mounted with Cytoseal mounting medium on glass slides, and the podocytes were observed using a confocal microscope (LSM-510 Meta; Carl Zeiss, Jena, Germany).

### Transmission electron microscopy

Paraformaldehyde-fixed kidney samples were embedded in epoxy resin. The processed samples were analyzed by electron microscopy (JEM-1400, JEOL, Tokyo, Japan).

### Western blotting and immunoprecipitation

Harvested cells or kidney tissues were lysed in buffer containing 50 mM Tris–HCl (pH 7.4), 150 mM NaCl, 1 mM EDTA, 1 mM Na_3_VO_4_, 20 mM NaF, 10 mM glycerophosphate, 1 mM PMSF, 10% glycerol, 1% TX-100, 0.2% SDS, and protease inhibitor cocktail. The samples were centrifuged and supernatants were analyzed. For immunoprecipitation, 0.5- to 1-mg aliquots of cell extracts were incubated with 2 μg of the indicated antibodies for 8 h at 4 °C under gentle agitation. Immunocomplexes were collected using protein A-Sepharose beads. Whole cell lysates or immunoprecipitates were subjected to sodium dodecyl sulfate-polyacrylamide gel electrophoresis (SDS-PAGE) and immunoblotting.

For immunoblotting, the lysates mixed with a 5X sample buffer containing 60 mM Tris-HCl (pH 6.8), 25% glycerol, 2% sodium dodecyl sulfate (SDS), 0.1% bromophenol, 0.2% 2-mercaptoethanol, and heated at 95 °C for 10 min. The lysates were separated on SDS-PAGE, transferred to nitrocellulose membrane, and probed using various antibodies. The blots were reacted with secondary antibodies followed by enhanced chemiluminescence (ECL system; Thermo Fisher, Waltham, MA, USA). Positive immunoreactive bands were quantified by densitometry and compared with those of the control.

### Statistical analysis

All results are expressed as mean ± standard error of the mean (SEM). Experimental data were compared using an unpaired two-tailed Student’s t tests or analysis of variance (ANOVA) with Tukey’s post-hoc analysis. P < 0.05 was considered significant.

## Electronic supplementary material

Supplementary information
