# Supplementary material for: C1-Ten is a PTPase of nephrin, regulating podocyte hypertrophy through mTORC1 activation
Source: Sci Rep. 2017 Sep 27;7:12346. doi: 10.1038/s41598-017-12382-8 (PMC5617844; doi:10.1038/s41598-017-12382-8)

C1-Ten is a PTPase of nephrin, regulating podocyte hypertrophy through mTORC1 activation

Authors: Jiyoun Lee[1], Ara Koh[1], Heeyoon Jeong[1], Eui Kim[2], Tae-Sun Ha[3], Moin A. Saleem[4], Sung Ho Ryu[1,*]

Affiliations:

1Department of Life Sciences, Pohang University of Science and Technology, Pohang, 37673, Republic of Korea

2Division of Integrative Biosciences and Biotechnology, Pohang University of Science and Technology, Pohang, 37673, Republic of Korea

3Department of Pediatrics, College of Medicine, Chungbuk National University, Cheongju, 28644, Republic of Korea

4Academic and Children's Renal Unit, University of Bristol, Learning and Research, Southmead Hospital, Bristol BS10 5NB, UK

*Corresponding author at Department of Life Sciences, Pohang University of Science and Technology (POSTECH), Pohang, 37673, Republic of Korea. Tel: 82-54-279-2292. Fax: 82-54-279-0645.

E-mail address: sungho@postech.ac.kr.

Supplementary Figure legends

Supplementary Fig. S1. High glucose mediates podocyte permeability defect with nephrin dephosphorylation. (a) Expression level of C1-Ten in the kidney of streptozotocin (STZ)-treated rats. Streptozotocin (100 mg/kg body weight) was dissolved in 0.1 mol/L citrate buffer (pH 4.5) and injected in 6-week-old male Sprague-Dawley rats by the tail vein. Control rats were injected with an equivalent volume of citrate buffer alone. Three days later, the rats were anesthetized and the kidneys were harvested. Expression levels of C1-Ten were measured by Western blotting in whole kidney lysates of control (Con) and STZ-treated rats. Data are means ± SEM (n = 3 rats per group). (b) Effect of high glucose (HG) on podocyte albumin influx in transwell permeability assay. Fully differentiated human podocytes were serum-starved for 18 h, then incubated in medium with normal glucose (NG) or HG for 24 h. Albumin concentration was evaluated using a Bradford assay at various time points. Data are means ± SEM (n = 3). (c) HG mediated reduction of nephrin phosphorylation. Fully differentiated human podocytes were incubated in medium with NG or HG for 24 h. Cell lysates were subjected to immunoprecipitation with anti-nephrin antibodies; immunoprecipitates were analyzed by immunoblotting with phosphotyrosine (pY), PI3K regulatory subunit α (p85α), and nephrin antibodies. (d) Fyn expression and Fyn-induced nephrin phosphorylation were confirmed by Western blotting in HEK293 cells. *, $P < 0.05$; **, $P < 0.01$.

Supplementary Fig. S2. Effect of nephrin on the basal mTORC1 signaling. HEK293 cells were transfected with GFP vector or GFP nephrin WT for 48 h. mTORC1 activation was measured by immunoblotting of phospho- and total- S6K1.

Supplementary Fig. S3. Additive effect of C1-Ten depletion and rapamycin on the HG-mediated podocyte hypertrophy. Human podocytes were transfected with 50 nM of control or TNS2 siRNA (siC1-Ten), then stimulated with HG for 24 h. Rapamycin (10 µM) was applied as a positive control under HG for 1 h. (a) Podocytes were stained with Alexa 594 phalloidin to observe morphology of cells (Scale bar, 100 µm). (b) Protein/number of podocytes also were evaluated using hemocytometer and a modified Lowry assay. Data are means ± SEM (n = 3). *, $P < 0.05$ vs. HG.

Supplementary Fig. S4. Uncropped images of Western blots.

Supplementary Figures

Lee J et al., Supplementary Fig. S1

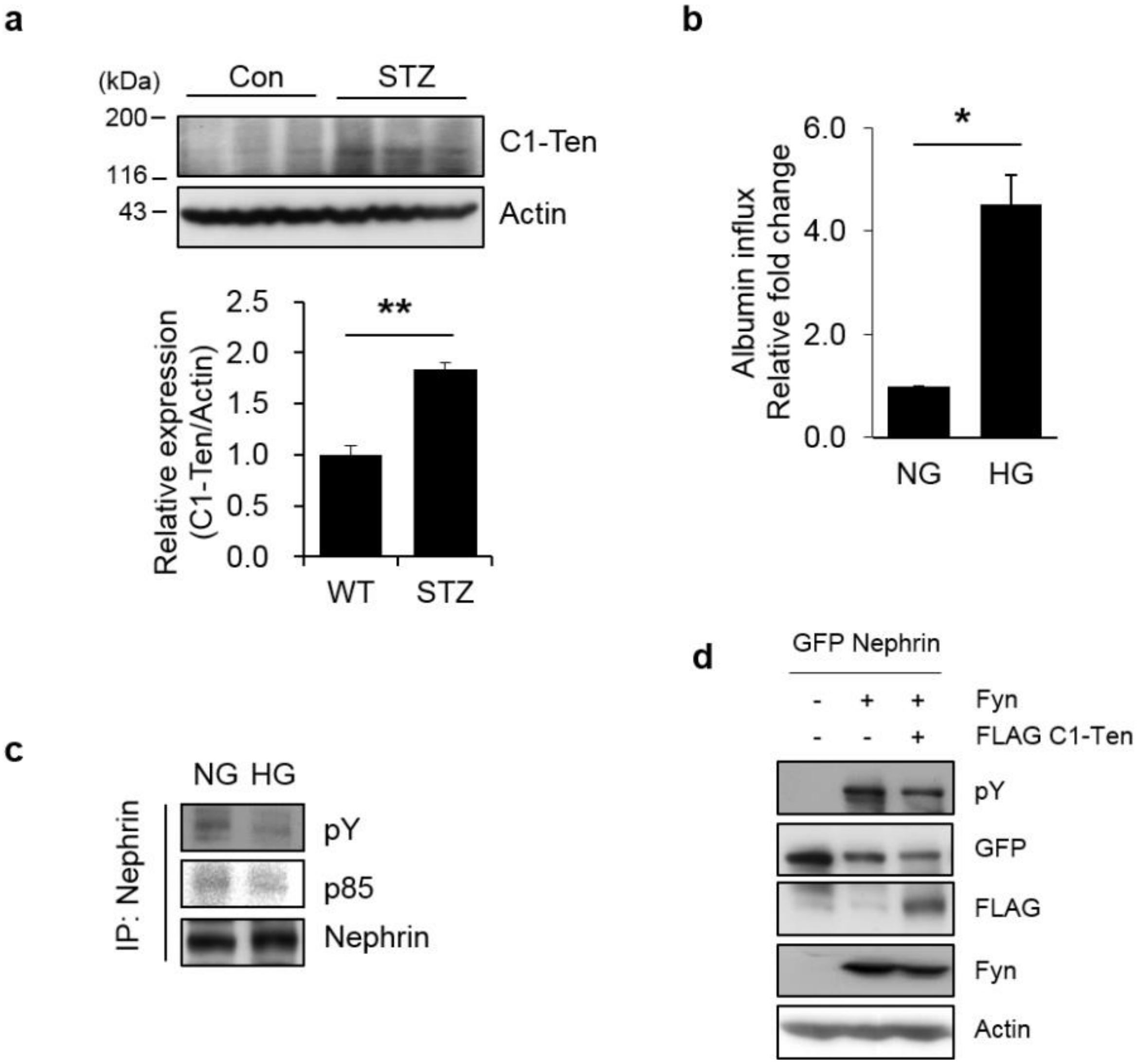

Lee J et al., Supplementary Fig. S2

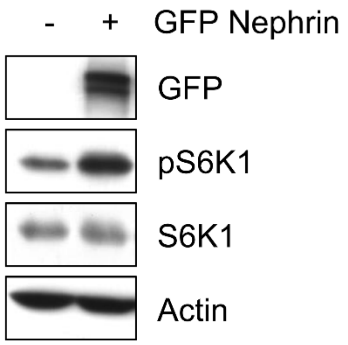

Lee J et al., Supplementary Fig. S3

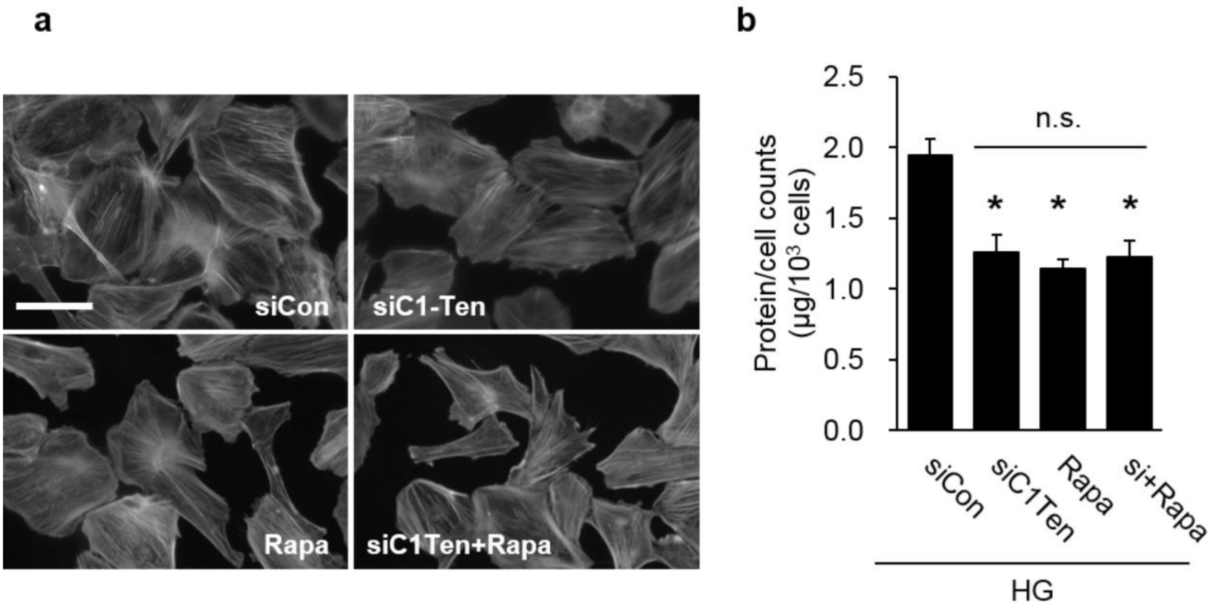

Lee J et al., Supplementary Fig. S4

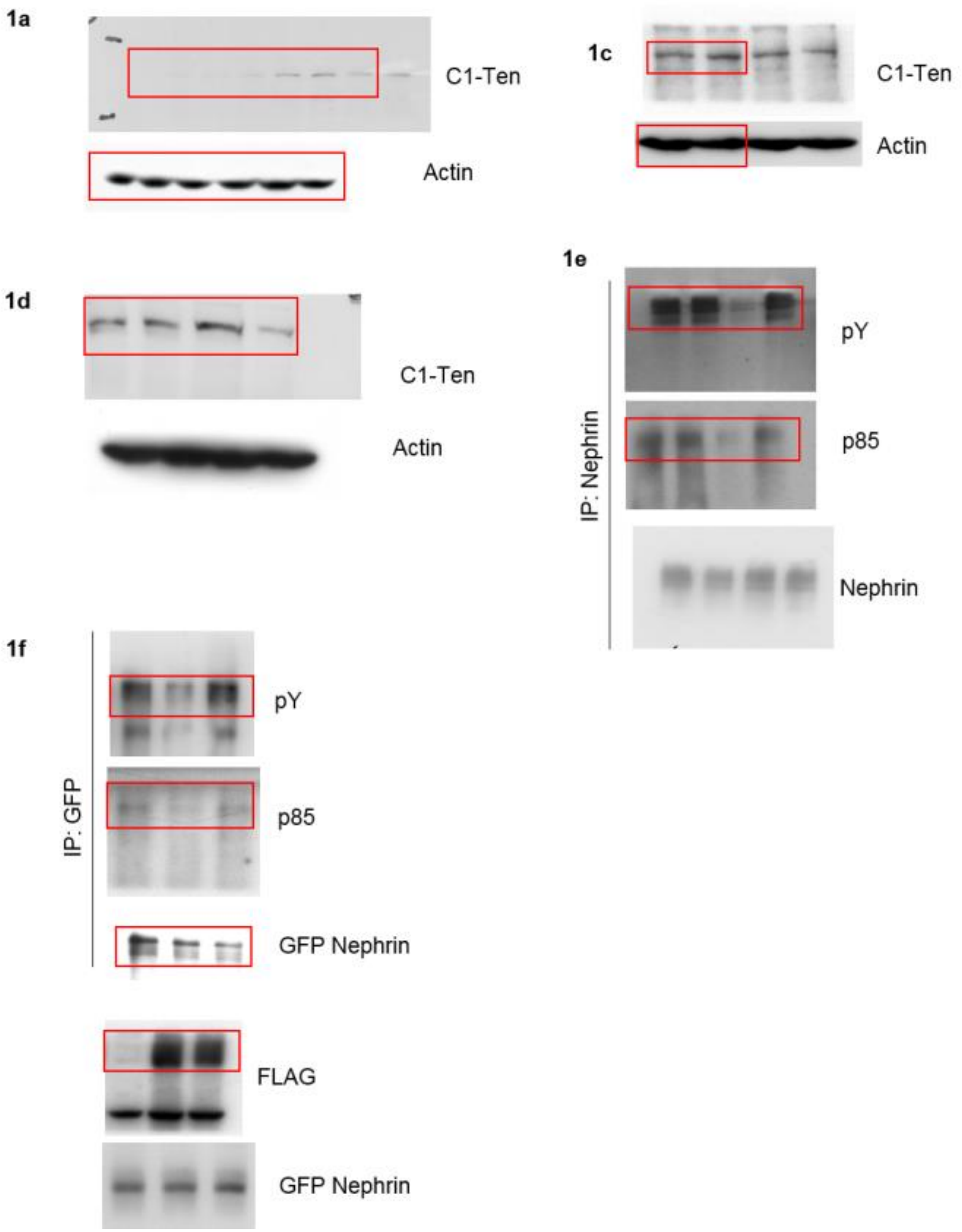

(continued)

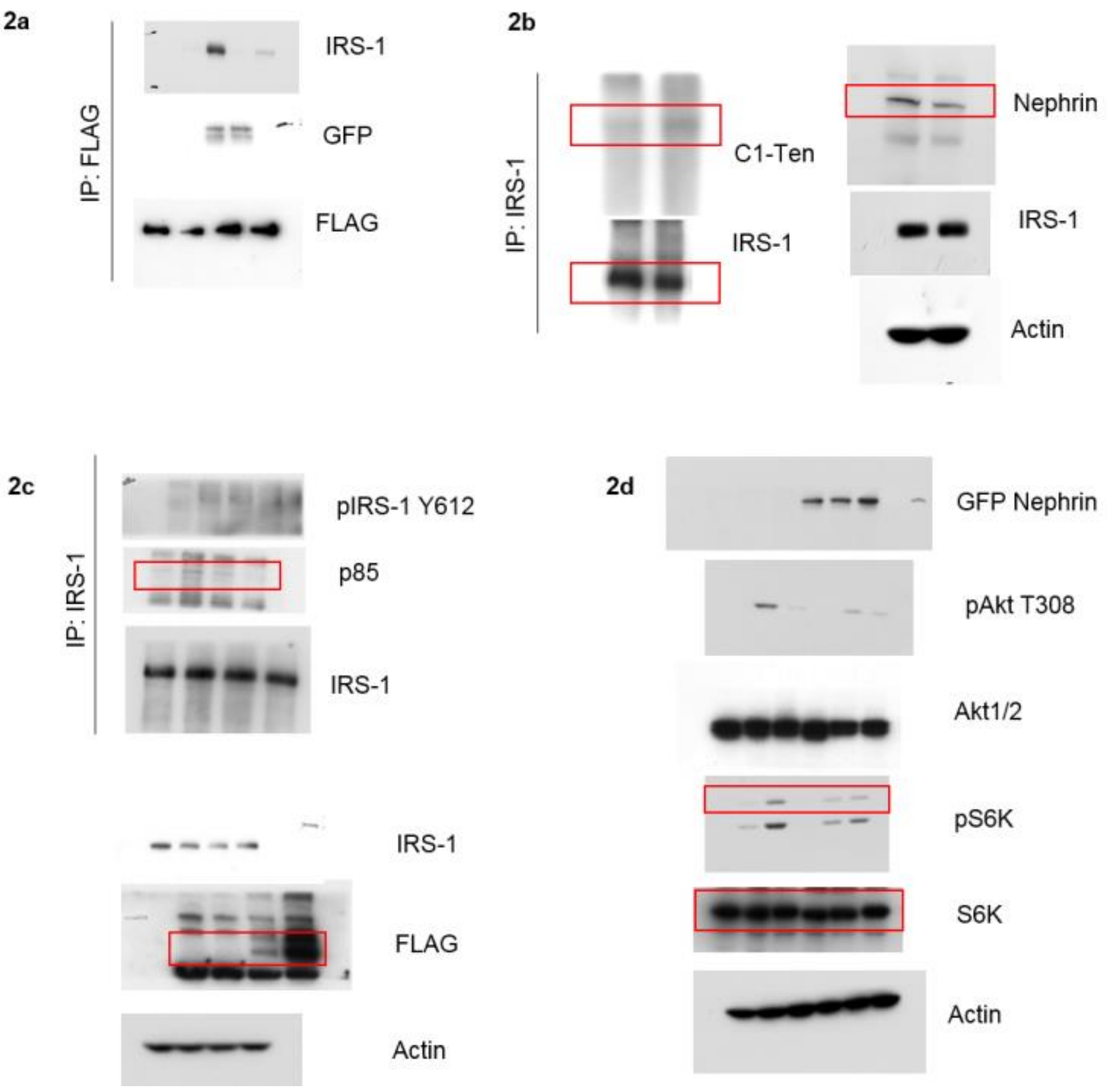
2a
IP: FLAG
IRS-1
GFP
FLAG
2b
IP: IRS-1
C1-Ten
IRS-1
Nephrin
IRS-1
Actin
2c
IP: IRS-1
pIRS-1 Y612
p85
IRS-1
IRS-1
FLAG
Actin
2d
GFP Nephrin
pAkt T308
Akt1/2
pS6K
S6K
Actin

(continued)

3a

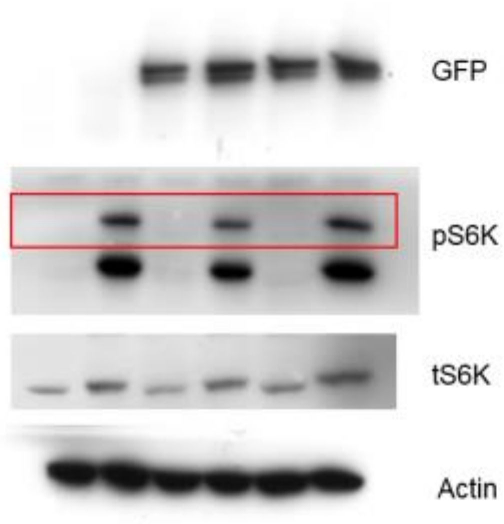

3b

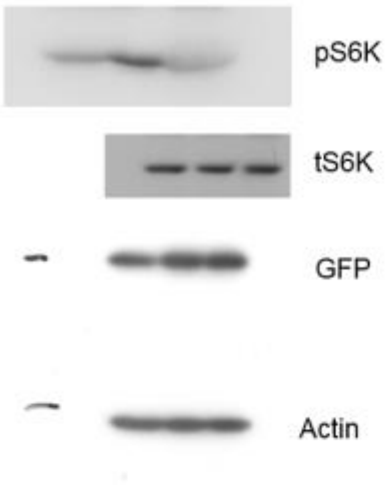

3c

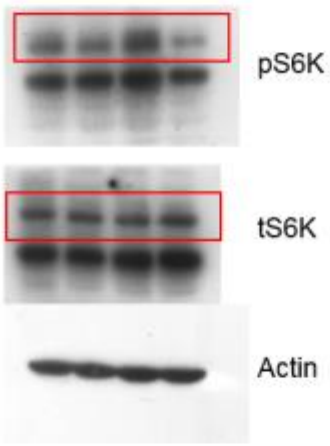

(continued)

5a

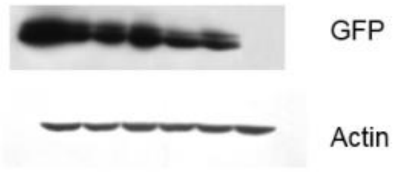

5c

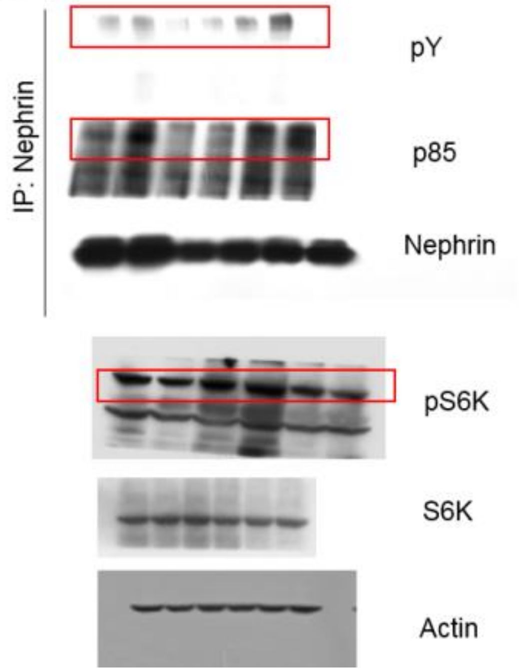

Supplement: Supplementary file 1 — Supplementary information [file 41598_2017_12382_MOESM1_ESM.pdf]
